# Functional Foods and Nutraceuticals for the Management of Cardiovascular Disease Risk in Postmenopausal Women

**DOI:** 10.31083/j.rcm2512460

**Published:** 2024-12-25

**Authors:** Harshini Meegaswatte, Kathryn Speer, Andrew J. McKune, Nenad Naumovski

**Affiliations:** ^1^Faculty of Health, University of Canberra, 2617 Bruce, Canberra, ACT, Australia; ^2^Functional Foods and Nutrition Research (FFNR) Laboratory, University of Canberra, 2617 Bruce, Canberra, ACT, Australia; ^3^University of Canberra Research Institute for Sport and Exercise (UCRISE), University of Canberra, 2617 Bruce, Canberra, ACT, Australia; ^4^Discipline of Biokinetics, Exercise and Leisure Sciences, School of Health Science, University of KwaZulu-Natal, 4041 Durban, Republic of South Africa; ^5^Department of Nutrition and Dietetics, School of Health Science and Education, Harokopio University, 17676 Athens, Greece

**Keywords:** cardiovascular disease, menopause, postmenopausal women, metabolic syndrome, vascular health, functional foods, nutraceuticals

## Abstract

Cardiovascular disease (CVD) is a leading cause of death in women and risk of development is greatly increased following menopause. Menopause occurs over several years and is associated with hormonal changes, including a reduction in estradiol and an increase in follicle-stimulating hormone. This hormonal shift may result in an increased risk of developing abdominal adiposity, insulin resistance, dyslipidemia, vascular dysfunction, hypertension, type 2 diabetes mellitus (T2DM), metabolic dysfunction-associated fatty liver disease (MAFLD), and metabolic syndrome (MetS). Furthermore, with the onset of menopause, there is an increase in oxidative stress that is associated with impaired vascular function, inflammation, and thrombosis, further increasing the risk of CVD development. Despite the harmful consequences of the menopause transition being well known, women in premenopausal, perimenopausal, and postmenopausal stages are unlikely to be enrolled in research studies. Therefore, investigations on the prevention and treatment of cardiovascular and metabolic disease in middle-aged women are still relatively limited. Whilst lifestyle interventions are associated with reduced CVD risk in this population sample, the evidence still remains inconclusive. Therefore, it is important to explore the effectiveness of early intervention and potential therapeutic approaches to maintain cellular redox balance, preserve endothelium, and reduce inflammation. Glycine, N-acetylcysteine, and L-theanine are amino acids with potential antioxidant and anti-inflammatory activity and are identified as therapeutic interventions in the management of age-related and metabolic diseases. The benefits of the intake of these amino acids for improving factors associated with cardiovascular health are discussed in this review. Future studies using these amino acids are warranted to investigate their effect on maintaining the vascular health and cardiovascular outcomes of postmenopausal women.

## 1. Introduction

Cardiovascular disease (CVD) is the leading cause of death globally and this increase is linked with lifestyle changes, and a growing and aging global population. Moreover, CVD is the leading cause of death in women [1, 2]. The risk of CVD in women increases after menopause and they may develop coronary heart disease several years later than men [3]. Menopause is a ‘*natural*’ biological process that progresses with aging, and it is characterized by the complete discontinuation of the menstrual cycle resulting from the cessation of ovarian function [4]. The process of menopause occurs over several years and starts with the onset of an irregular menstrual cycle in women, which is defined as menopausal transition or perimenopause [5]. Menopause transition leads to a reduction in circulating hormones such as estradiol (E2) and an increase in follicle-stimulating hormone (FSH) [6].

**Fig. 1.**
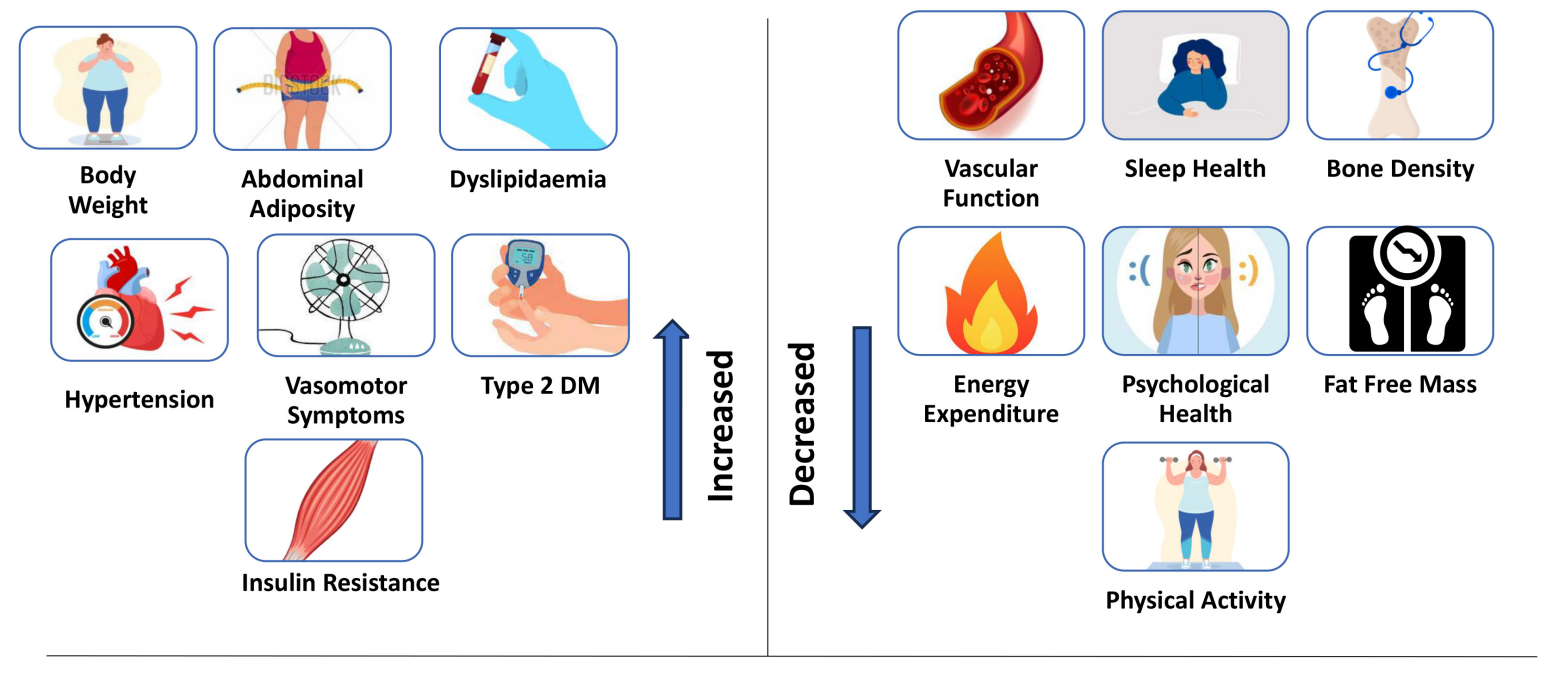
**Metabolic, psychological, physical changes across the menopause transition associated with the increased risk of cardiovascular disease (CVD) development in postmenopausal women**. DM, diabetes mellitus.

Sex hormones also impact body fat distribution, adipocyte differentiation, and the association between the increased overweight/obesity in women during menopause and hormonal changes in the menopause transition are well established [7]. The weight gain in menopause particularly increases abdominal adiposity contributing to cardiovascular risk [8]. The menopausal transition increases the risk of insulin resistance, dyslipidemia, vascular dysfunction, and hypertension, consequently leading to individuals developing type 2 diabetes mellitus (T2DM), metabolic dysfunction-associated fatty liver disease (MAFLD), metabolic syndrome (MetS), and CVD [9, 10, 11]. Furthermore, MetS is characterized by increased oxidative stress and inflammation [12] due to the reduced activities of plasma antioxidant enzymes and increased production of reactive oxygen and nitrogen species [13, 14]. This increased oxidative stress may contribute to the pathogenesis of MetS by promoting inflammation, thrombosis, and atherosclerosis, further increasing the risk of the development of CVD in women [15, 16]. In addition, psychological health (mood swings, anxiety, depression) and physical health (sleep health, bone density, balance) are also negatively affected during the menopausal transition and associated with increased CVD risk [17, 18]. Fig. 1 illustrates the metabolic, psychological, and physical changes manifest across the menopause transition associated with the increased CVD risk [8, 9, 17, 18].

Although these harmful consequences of menopause transition are well known, women in premenopausal, perimenopausal, and postmenopausal stages are unlikely to be enrolled in CVD research study [19]. The onset and development of CVD in women is still relatively understudied, under-recognized, underdiagnosed, and undertreated posing a significant health burden in women that has stagnated over the past decade [20]. Therefore, it is important to understand the relationship between the menopause transition and CVD risk in post-menopausal women for the early detection of cardiovascular health changes relevant to menopause transition, treatment, and management of it to decrease CVD morbidity and mortality in post-menopausal women.

Lifestyle modifications, such as dietary interventions, play a significant role in the prevention of CVD [21]. Furthermore, the dietary intake of postmenopausal women is associated with the severity of the menopausal symptoms including psychological symptoms, somatic symptoms, vasomotor symptoms, sleep disorders, and urological symptoms [22]. Though research studies have been conducted to explore the effects of supplements, nutrients, isolated single foods, and whole diets on menopausal health issues in postmenopausal women [22, 23, 24, 25], the findings remain inconclusive.

There is a scarcity of studies conducted to investigate the effect of functional foods and nutraceuticals in the management of cardiovascular risk in postmenopausal women. Thus, the purpose of this narrative review is to explore the cardiovascular health and modifiable risk factors of cardiovascular disease in postmenopausal women and to discuss potential dietary interventions using functional food and nutraceuticals for enhancing their cardiovascular health.

## 2. Menopause Transition 

Menopause is characterized by the permanent discontinuation of menstruation caused by the reduction of ovarian follicles which is generally defined by the final menstrual period with a median age of approximately 51 years [26]. It is also associated with a decline in physical and psychological health [9] and the menopause transition is the time from the beginning of the menstrual cycle changes up to one year after the final menstrual period which usually starts around 47 years [27]. The perimenopause period includes the menopausal transition with the initial sign of ovarian function depletion up to one year from the final menstruation [28]. Menopause accelerates the rate of ovarian follicle deterioration which leads to a drastic reduction in the circulating estrogen specifically the E2 level, and a rise in the gonadotropin FSH levels [6]. The ovaries are stimulated by the elevated FSH to produce E2, resulting in a further increase of FSH as the ovarian function decreases [29]. The ovaries cannot secrete E2 in response to the elevated FSH whereas the ovarian follicle contribution reaches an extremely low level throughout the transition from early to late perimenopause. Therefore, the elevated FSH level remains stable when the E2 production discontinues and is used as a diagnostic tool to distinguish between early and late perimenopause [27].

## 3. Cardiovascular Health 

Estrogen has a positive effect on inflammation, oxidative stress, the production of nitric oxide (NO) by vascular endothelium, and the remodeling of the vasculature in women. Vascular aging, which indicates the continuing stiffening of arteries and reduction in vasodilatory capacity, accelerated simultaneously with the onset of menopause [30, 31]. Reduced estrogen production with menopause may be responsible for accelerated vascular aging [32] that involves endothelial cell dysfunction, vascular remodeling, increased vascular stiffness, and inflammation. Increased vascular aging contributes to a greater prevalence of hypertension and other risk factors for the development of CVD in postmenopausal women [33, 34, 35]. A study by Moreau *et al*. [36] (2012) reported that vascular endothelial function starts to decrease in the perimenopausal period and exacerbates during the postmenopausal period, independent of age, CVD risk factors, physical activity, and aerobic capacity.

The endothelium contributes significantly to regulating vascular function by secreting molecules involved in vasodilation and vasoconstriction [34, 37]. The main vasodilator produced by the endothelium is NO, which is synthesized from the amino acid L-arginine in the presence of endothelial nitric oxide synthase (eNOS), facilitated by the co-factor tetrahydrobiopterin [38]. Reduction in NO bioavailability can occur due to the decreased NO synthesis and increased NO inactivation by reactive oxygen species (ROS), making the depleted NO levels as a potential causative factor for the pathogenesis of endothelial dysfunction [39, 40]. Moreover, an imbalance of the vasodilating and vasoconstricting molecules produced from the endothelium is caused by the continuing dysfunction of the endothelial cell layer [41].

**Fig. 2.**
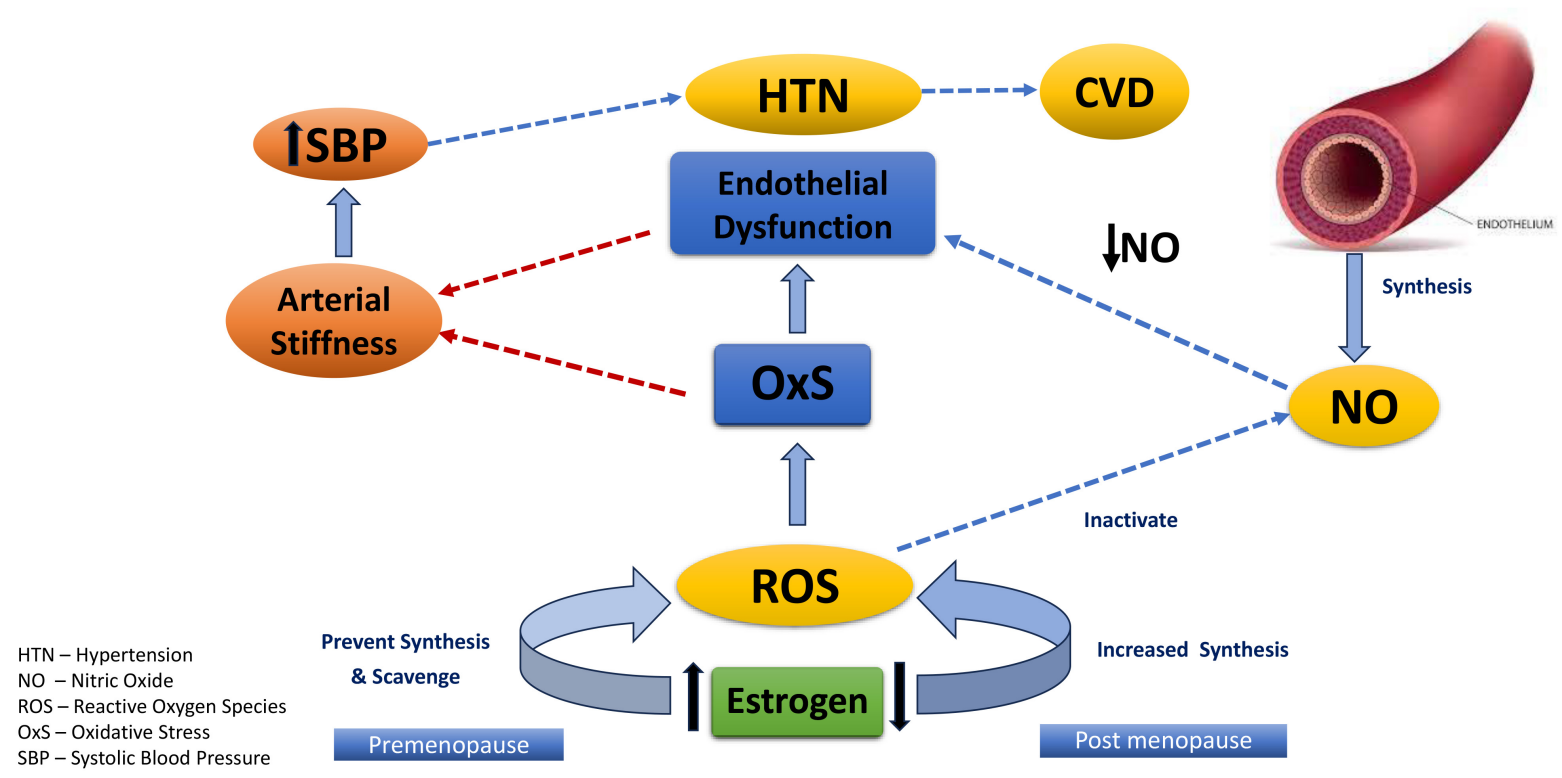
**Changes in vascular function with the estrogen hormone reduction during the menopause transition**. CVD, cardiovascular disease.

Oxidative stress is the imbalance between the production and degeneration of the ROS and/or reactive nitrogen species [42]. Ovarian estrogen is considered to be involved in the inhibition of ROS production as well as ROS scavenging due to its potential antioxidant capacity [43]. Estrogen reduction in postmenopausal women results in decreased ROS production inhibition which leads to a change in the redox balance and an increase in oxidative stress. Consequently, this increase in oxidative stress may lead to endothelial dysfunction and damage to lipids, proteins, and deoxyribonucleic acid (DNA) in perimenopausal and post-menopausal women [44, 45, 46, 47]. Endothelial dysfunction and oxidative stress are associated with the progression of arterial stiffness that further leads to the elevation of systolic and pulse pressure which are directly linked with hypertension [30, 48, 49]. Fig. 2 illustrates the changes in vascular function with the estrogen hormone reduction in menopause transition [32, 39, 42, 45, 46, 47, 48, 49, 50, 51, 52, 53, 54, 55, 56].

It remains unclear whether the decreased endothelial function with the menopausal transition is due to the change in sex hormone level or the change in ovarian function [45]. Furthermore, the clinical significance of oxidative stress, endothelial dysfunction, and inflammation in postmenopausal women during the menopause transition is associated with increased CVD risk [36]. Therefore, it is important to develop a potential therapeutic approach to preserve the endothelium and maintain cardiovascular health in postmenopausal women.

## 4. Changes in Metabolism and Metabolic Syndrome in Postmenopausal Women

Menopause also increases abdominal adiposity, changes in lipid profile, and development of insulin resistance [15, 50]. Longitudinal cohort studies have revealed that menopause transition also involves a transition from an estrogenic to an androgenic state due to an increase in circulating androgens such as dehydroepiandrosterone (DHEA), its metabolite DHEA sulphate (DHEAS), androstenedione, testosterone, and dihydrotestosterone (DHT). DHEA, DHEAS, and androstenedione need to be converted to testosterone to utilize its androgenic effect, thus being considered as pro-hormones [51, 52, 53]. The visceral adipose tissue secretes adipocytokines, proinflammatory cytokines, ROS, prothrombotic, and vasoconstrictor factors associated with further weight gain, hyperlipidemia, hypertension, and insulin resistance [15, 54].

MetS is not a disorder, but rather a cluster of factors including abdominal obesity, dyslipidemia, hyperglycemia, insulin resistance, and hypertension that further contribute to the increased risk of developing CVD [15]. Prevalence of MetS is higher in men compared with same-age premenopausal women, which reverses during menopause resulting in a higher prevalence of MetS in women [55, 56]. According to The Study of Women’s Health Across the Nation (SWAN), the prevalence of MetS increased continuously from six years before and six years later to the final menstrual period independent of age and known CVD risk factors [57].

### 4.1 Weight Gain and Insulin Resistance in Postmenopausal Women 

Menopause transition is commonly associated with up to 3 kg weight gain, although there is large interpersonal variability between different ethnic groups [58]. The changes in body composition (increased fat mass, decreased fat-free mass, and bone mineral density) may occur without leading to weight gain in some women [19]. The findings from the SWAN study suggested an accelerated gain in fat mass and loss of fat-free (lean) mass during the menopause transition [59]. Moreover, E2 plays an important cardio-protective role by contributing to both insulin sensitivity and peripheral fat distribution in premenopausal women [60]. Loss of E2 function throughout the menopause transition resulted in abdominal (central) adiposity and the development of insulin resistance, increasing the risk of developing T2DM in postmenopausal women [61]. Menopause transition may also result in decreased fat oxidation and a reduction in resting energy expenditure despite any alterations in energy intake [62]. The presence of 36% extra thoracic fat and 49% higher intra-abdominal fat in postmenopausal women compared with premenopausal women was shown to be, independent of their age and total fat mass [63]. Postmenopausal women had up to 50% increased insulin resistance and 50% reduced pancreatic insulin secretion compared with premenopausal women regardless of body mass index (BMI) and age [64]. Insulin resistance and dyslipidemia have also been shown to increase the influx of fatty acids to the liver and increase the development of MAFLD in postmenopausal women [65].

### 4.2. Lipid Metabolism Disorders 

Dyslipidemia in menopause is associated with a reduction in estrogen during menopausal transition. The findings of the SWAN study proposed that there is an increase in total cholesterol (TC), low-density lipoprotein (LDL)-cholesterol (LDL-C), and apolipoprotein B (ApoB) levels within one year before and one year after the final menstrual period. A decline in high-density lipoprotein (HDL)-cholesterol (HDL-C) was also observed during the menopause transition, which had been shown to increase CVD risk in postmenopausal women [9, 57]. Higher HDL-C levels are associated with low carotid atherosclerosis before menopause and higher carotid atherosclerosis after menopause [66]. Therefore, the change from a primarily estrogenic to an androgenic condition during the menopausal transition leads to metabolic abnormalities culminating in MetS in postmenopausal women [54].

## 5. Sleep Health in Postmenopausal Women

It is well established that the maintenance of high-quality sleep is necessary for healthy aging [67]. Regular sleep complaints are reported in older adults and are usually associated with physical, environmental, and health factors such as an increase in mortality, development of obesity, T2DM, and CVD [68]. In general, sleep differs between males and females across the lifespan. Decreases in estrogen and progesterone and changes in the ratio of estrogen to testosterone during the menopause transition are associated with sleep disturbances [69]. These disturbances result in deleterious physical and psychological symptoms [18]. Specific sleep disturbances commonly observed in menopause are insomnia, poor sleep quality, sleep deprivation, excessive daytime sleepiness, and sleep disorders such as obstructive sleep apnea, restless legs syndrome, depression, and several different mood and anxiety-related disorders [70]. Higher levels of sleep disturbances and a high risk of clinical insomnia can be seen in women who are experiencing vasomotor symptoms [71]. Disturbed sleep quality is also associated with poor quality of life, increased energy intake, intake of high-fat and high-sugar foods, decreased physical activity, weight gain, and obesity risk [68].

The prevalence of sleep disturbance varies from 16% to 42% in the premenopausal age group, 39% to 47% in perimenopausal women, and between 35% to 60% in postmenopausal women [72]. A higher incidence of sleep disorders in menopause mediates the association between fibromyalgia, perimenopause, and surgical menopause in women [73]. Furthermore, menopausal sleep disorders can be an independent risk factor for increased arterial stiffness in menopause, which increases CVD-related morbidity and mortality [74]. Therefore, postmenopausal women have an increased risk of sleep disturbances as well as sleep disorders, making this area an opportunity to explore potential therapeutic interventions to preserve the overall quality of life in postmenopausal women.

## 6. Methods

In May 2023, a nonsystematic literature search was conducted using the Google Scholar electronic database to screen papers with the following search strategy; “functional food” or “nutraceuticals”, “dietary intervention”, “N-acetylcysteine”, “glycine”, “L-theanine”, “glutathione”, “cardiovascular health”, “cardiovascular disease”, “vascular function”, “endothelial dysfunction”, “hypertension”, “postmenopausal women”, “menopause transition”, “menopause”, “metabolic syndrome”. Articles included in this review were required to be in English and the clinical studies conducted with functional foods or nutraceuticals with postmenopausal women or adults ages 18 years and over were included in the review. Moreover, the preclinical studies included animal, human and *in vitro* conducted with proposed functional foods and nutraceuticals were also included in the review. The same searches were also performed prior to submission of the final manuscript. Based on the information obtained from publications, selected journal articles were used in this review.

## 7. Potential Functional Foods and Nutraceuticals in the Management of Cardiovascular Health in Postmenopausal Women

The safety and benefits of hormone replacement (HRT) therapy in postmenopausal women have been debated and the use of HRT by postmenopausal women has declined because of the contradictory findings of clinical trials which reported that HRT was associated with health risks, primarily reduced cardiovascular health, and increased incidence of breast cancer [75]. Therefore, the use of non-hormonal, pharmacological, and nonpharmacological interventions has increased as an alternative to HRT in the management of postmenopausal symptoms [76]. Lifestyle modifications contribute to the reduction of CVD risk in postmenopausal women [77]. Improving diet is one of the most significant modifiable factors for the prevention of CVD [21]. Dietary intake of postmenopausal women has been shown to be associated with the severity of the menopausal symptoms including psychological, somatic, and vasomotor symptoms, and sleep disorders [22, 78, 79, 80].

Understanding the impact of nutrition on health and well-being has increased during the last two decades consequently leading to the development of novel and ‘*healthier*’ food products [81]. Functional foods are defined as food products that have an addition of a functional ingredient that may exhibit beneficial health outcomes [82]. The intake of these food products has seen a rapid increase prompting more research in the analysis of the potential health benefits of functional foods [81]. With scientific advancement, research in the area of nutrition in combination with pharmaceutical sciences and human health has resulted in innovative new dietary products termed nutraceuticals. Nutraceuticals are foods, or its products, that have health improvement characteristics and micronutrients such as vitamins, minerals, amino acids, and probiotics belong to this category [83]. Furthermore, bioactive composites available in different foods and their products were shown to have a potential positive effect on cardiovascular health *via* epigenetic mechanism [84, 85]. Some of the *in vitro* work and a clinical study has shown that functional foods regulate the effectors of cardioprotective gene expression [84]. Furthermore, exosomes, mini extracellular vesicles which enable cell to cell communication are being identified as a cardioprotective agents and were reported to be available in some foods such as lemon, ginger, milk, broccoli, grapes, grapefruit and carrots [86, 87, 88]. However, most of the studies conducted in exosomes are *in vitro* cell-based assays or animal model studies and the interpretation of the findings from these studies towards the human applicability should be taken with caution [87].

However, the findings from studies conducted to investigate the effects of supplements, nutrients, isolated single foods, and whole diets on overall menopausal health issues are listed in Table 1 (Ref. [75, 76, 89, 90, 91, 92, 93, 94, 95, 96, 97, 98, 99, 100]) and those findings in postmenopausal women are conflicting and inconclusive [17, 18, 19, 20].

**Table 1.** **Summary of studies using functional foods, nutraceuticals, isolated single foods, and whole diets on overall menopausal and post-menapausal health**.

Design/Randomization	No. of participants/interventions	Health status	Intervention functional food/Nutraceutical	Control	Duration	Results	Reference
Randomized, double-blind, placebo-controlled, parallel design study	44 postmenopausal women	Healthy	6 g of L-Citrulline	Placebo	4 weeks	Does not improve brachial artery FMD	[76]
			2 g of L-Citrulline + 200 mg of GSH	Placebo	4 weeks	Improve brachial artery FMD	
Randomized, double-blind, placebo-controlled, parallel-design study	24 postmenopausal women	Hypertensive and sedentary	10 g L-Citrulline	Placebo	4 weeks	Improve leg endothelial function	[89]
			10 g L-Citrulline + SVLIRT	Placebo + SVLIRT	4 weeks	Benefits on leg LM and curl strength	
Randomized, double-blind, placebo-controlled, parallel-design study	25 postmenopausal women	Hypertensive and sedentary	10 g L-Citrulline	Placebo	4 weeks	Improve endothelial function and aortic BP via increased L-ARG availability	[90]
Randomized, double-blind, placebo-controlled, crossover study	20 postmenopausal women	Healthy	NO_3_^−^ rich (BR _nitrate_) Beetroot juice consumption	NO_3_^−^ depleted (BR _placebo_)	Single dose	Minimize IR-induced macrovascular endothelial dysfunction, does not improve resting macrovascular and microvascular function	[91]
Randomized, double-blind, placebo-controlled, clinical trial	20 postmenopausal women	Metabolic syndrome	Genistein 54 mg/day (two tablets/day)	Placebo	6 months	Improved endothelial function and FMD	[92]
Randomized, double-blind, placebo-controlled, parallel-arm study	32 postmenopausal women	Healthy and sedentary	Curcumin group—Curcumin 150 mg/day (6 pills) aerobic	Placebo	8 weeks	Flow-mediated dilation increased significantly and equally in the curcumin and exercise groups, whereas no changes were observed in the control group	[93]
			Exercise group—exercise training more than 3 days per week				
Meta-analysis of randomized placebo-controlled trials	Postmenopausal women	Healthy	Isoflavones		2–12 weeks	Does not improve endothelial function in postmenopausal women with high baseline FMD levels but leads to significant improvement in women with low baseline FMD levels	[94]
			(50 mg–25 g/day)				
Systematic review and meta-analysis of randomized controlled trials	Postmenopausal women		Soy protein supplementation	Placebo		No significant improvement in FMD	[95]
Meta-analysis of randomized controlled trials	Postmenopausal women		Phytoestrogen supplementation	Placebo		Moderately reduced CVD risk through improving blood lipids and endothelial function and shown to have a detrimental effect on carotid intima-media thickness, increasing the risk of atherosclerosis	[96]
Randomize double-blind placebo-controlled clinical trial	244 postmenopausal women	Healthy	Menaquinone	Placebo	3 years	Increased arterial stiffness in women with higher baseline stiffness	[97]
			(180 µg/day)				
Randomized crossover design	23 postmenopausal women	T2DM	Fish-based diet	Control diet	8 weeks	Endothelial function and lipid metabolism improved and no relationship between n-3 PUFA and endothelial function	[98]
Randomized, double-blind, placebo-controlled study	60 postmenopausal women	Healthy	200 mg of fermented soy containing 10 mg of equol and 25 mg of resveratrol	Placebo	12 weeks	Dietary supplementation with equol and resveratrol may improve menopause-related quality of life	[99]
			(1 tablet/day)				
Pre-clinical study	Ovariectomized rats	Ovariectomized	α-lipoic acid (LA), docosahexaenoic acid (DHA) and eicosapentaenoic acid (EPA)		16 weeks	Alter enzymatic and non-enzymatic antioxidants in the livers of ovariectomized rats and DHA decreased protein and lipid damage	[100]
Pre-clinical study	Ovariectomized rats	Ovariectomized	N-acetylcysteine (NAC) and alpha-lipoic acid (LA)		Short- (15 days), long-term (60 days)	NAC and LA improve oxidative stress, cholesterol, and inflammatory components associated with sexual hormone depletion	[75]

Note: FMD, flow-mediated dilation; SVLIRT, slow velocity low-intensity resistance training; IR, ischemia-reperfusion; GSH, glutathione; LM, lean mass; L-ARG, L- arginine; CVD, cardiovascular disease; T2DM, type 2 diabetes mellitus; PUFA, polyunsaturated fatty acids; BR, beetroot juice; BP, blood pressure.

However, there is a considerable paucity of studies that investigate the effects of functional food on cardiovascular health, endothelial dysfunction, oxidative stress, and inflammation of postmenopausal women. Therefore, there is a need for further studies investigating the effectiveness of antioxidant and anti-inflammatory nutraceuticals and functional foods in the management of cardiovascular health in postmenopausal women. Specific nutraceuticals that have been shown to have positive impacts *via* the reduction of oxidative stress and inflammation include the amino acids N-acetylcysteine [101, 102, 103], glycine [104, 105, 106, 107, 108], and L-theanine [109, 110, 111, 112, 113].

### 7.1 N–acetylcysteine

N-acetylcysteine (NAC) is an acetylated cysteine compound extensively used in mucolytic therapy and to treat paracetamol overdose usually administrated orally, intravenously, or by inhalation [114]. The World Health Organization has accepted NAC as a pertinent medicine essential in the health care system [114] while it is designated as a nutritional supplement and is regularly accessible without prescriptions [101]. Predominately due to its antioxidant properties, supplementation with NAC has been identified as a therapeutic intervention for health conditions related to oxidative stress. NAC increases glutathione (GSH) synthesis and availability by acting as a precursor of cysteine, which is the rate-limiting step in GSH synthesis. The NAC also acts as a scavenger of ROS facilitating the cellular redox balance. Anti-inflammatory activity of NAC has been observed in some studies where NAC has controlled the release of cytokines at the beginning of immune proliferation and may inhibit tumor necrosis factor alpha (TNF-α) and interleukins (IL) such as IL-6 and IL-1β in humans [100, 101]. GSH is the most abundant, endogenous, intracellular antioxidant and is responsible for the defense mechanism against oxidative stress [103]. With prolonged exposure to oxidative stress, GSH levels can become depleted, resulting in decreased endogenous antioxidant defense ability, and the need for the generation of intracellular GSH from NAC [114]. Supplementing with NAC to increase circulating and intracellular levels of GSH has been proposed as a method to improve health across several different clinical populations [103, 114, 115].

The administration of NAC can be done orally, intravenously, or by inhalation [116]. Oral administration subjected to fast intestinal absorption and metabolism by the liver thus leading to its maximum plasma concentration between 1 and 2 hours [117, 118]. The unfavorable effects of NAC consumption may range from mild to severe depending on the formulation and dosage used and intravenous and oral NAC is related with minimal side effects [101]. A minimum effective dose of NAC is 9 mg/kg and the NAC doses used in interventions ranged from 9 mg/kg to 140 mg/kg [119]. The most common side effects in oral administration of NAC are gastrointestinal symptoms (nausea, vomiting) and other reactions such as itching and erythema [117, 120].

To the best of our knowledge, no clinical studies have investigated the impact of NAC supplementation on the management of cardiovascular health in postmenopausal women. A clinical trial reported that metformin intake and NAC supplementation lowered mammographic breast density in postmenopausal women. The potential mechanism was suggested to be *via *alterations in cell proliferation, apoptosis, and DNA repair rather than a metabolic effect such as lowered insulin resistance [121]. The NAC supplementation has also been shown to elevate the reduced intracellular GSH levels of leukocytes and improve immune function in postmenopausal women [122]. A pilot study showed that NAC supplementation reduced rapid bone resorption during the early postmenopausal years in women [123]. Furthermore, in animal models, a study investigated ovariectomized mice to determine the effects of FSH on menopausal depression. The findings showed that the depression-like behaviors of mice were decreased by NAC administration [124]. Another study in animals investigated the effect of NAC and alpha-lipoic acid supplementation on oxidative, inflammatory, and metabolic parameters in ovariectomized Wistar rats. The finding of this study reported that reduced sexual hormone levels, associated with oxidative stress, cholesterol, and inflammatory components, were improved with NAC and alpha-lipoic acid supplementation [75]. An *in vitro* study suggested a dose-related increase in the rate of cell proliferation and suppression of collagenase activity in cultured skin fibroblasts treated with resveratrol either alone or combined with different doses of NAC [125]. Therefore, NAC may have antioxidant and anti-inflammatory action in human and animal studies. However, there is a need to investigate the effect of NAC supplementation on the cardiovascular health of postmenopausal women.

### 7.2 Glycine

Glycine is an amino acid with the lowest molecular weight, a building block for protein synthesis [126] and essential in several metabolic mechanisms with a primary role in the synthesis of GSH and in one-carbon metabolism [106]. Glycine is a non-essential amino acid as it is produced endogenously but becomes conditionally essential when the endogenous synthesis of glycine is inadequate to fulfill the metabolic requirements [127]. The consumption of glycine has been associated with improvements in metabolic health by reducing oxidative stress and inflammation in humans [128].

Glycine can be safely administrated orally and intravenously and side effects with higher doses result in gastrointestinal symptoms (nausea, vomiting) [128]. Acute glycine supplementation is 5 g/day and longer administration can be given as 0.1 g of glycine/kg/day [129, 130]. A dose of 15 g of glycine per day is the highest tolerable dose in adult humans [107]. However, there is a need for studies to establish the optimum dosage, route, and intervention duration of glycine.

Though studies have investigated the effect of glycine on different health aspects of humans [106], only animal model studies have been conducted to investigate the effect of glycine on endothelial function and oxidative stress in post-menopause [131]. One study suggested that oxidative stress in postmenopausal women may be reduced by increased antioxidant action and improved lipid profile due to the consumption of yellow soybeans, black soybeans (Glycine max *L. Merr.*), or sword beans [132]. Endothelial dysfunction of ovariectomy rats was improved by soy isoflavones, but possibly without any changes in the reproductive system [133]. Moreover, soy isoflavones were used as a natural phytoestrogen which has estrogenic activity to reduce oxidative stress and insulin resistance in ovariectomized rats due to their antioxidant and anti-diabetic properties [131].

### 7.3 Glycine and N-acetylcysteine (GlyNAC) 

Intracellular mitochondrial dysfunction and oxidative stress are recognized as the primary factors in the aging process [134]. Thus, deficiencies in endogenous antioxidants, GSH, and its precursor amino acids glycine and cysteine may be responsible for the occurrence of oxidative stress and mitochondrial dysfunction [135]. Increasing intracellular levels of GSH cannot be achieved through oral supplementation with GSH since it is digested in the gut. Furthermore, each organ has its own variability in GSH concentrations and the administration of GSH will be difficult, although it’s delivered in a form that does not digest in the gut [113]. Moreover, GSH deficiency does not occur due to the depleted synthesis but primarily because of the reduced provision of the precursors glycine and cysteine [136]. Therefore, oral supplementation with glycine and cysteine in the form of NAC (GlyNAC) is seen to be more favorable for the production of GSH [136]. Recent human trials [137, 138, 139, 140, 141] and animal model [142, 143] studies have revealed that the consumption of GlyNAC may improve GSH levels and mitochondrial dysfunction including diminishing oxidative stress, inflammation, oxidation, insulin resistance, endothelial dysfunction, and genotoxicity [135]. Therefore, GlyNAC supplementation is a potential dietary supplement that may improve cardiovascular function and other menopause-related health complications.

Though the effect of GlyNAC on varied health outcomes of postmenopausal women has been investigated in animal models, to the best of our knowledge, there have been no human trials conducted to study the effect of GlyNAC on postmenopausal health outcomes. Furthermore, only a few studies have investigated the effect of GlyNAC on endothelial function [137, 138, 139]. However, a study by Kumar *et al*. [137] (2020) suggested that GlyNAC supplementation improved GSH deficiency, oxidative stress, mitochondrial dysfunction, inflammation, endothelial dysfunction, insulin resistance, genomic damage, and cognitive shortcomings resulting from premature aging in patients living with human immunodeficiency virus. Moreover, GlyNAC supplementation over 16 weeks was shown to be safe and well tolerated in older adults. Supplementation improved GSH deficiency, oxidative stress, mitochondrial dysfunction, inflammation, endothelial dysfunction, insulin resistance, and several aging hallmarks, such as impaired physical activity, elevated waist circumference, and systolic blood pressure [138]. These findings were further supported in a pilot clinical trial with older adults that examined the effect of 24 weeks of GlyNAC supplementation. The study found that GlyNAC supplementation could improve the cellular, mitochondrial, and metabolic health as well as the strength and cognition of older adults [139]. Furthermore, a study by Lizzo *et al*. [140] (2022) suggested that GlyNAC is safe and tolerable and may improve the GSH levels in older adults with higher GSH demand, but GSH levels continued to be the same in the older adults who have elevated oxidative stress [123]. Another pilot study on T2DM patients found that mitochondrial dysfunction, insulin resistance, and plasma fatty acid concentrations were improved by GlyNAC supplementation [124]. In animal models, a study conducted on rodents found that GlyNAC supplementation improved GSH deficiency, oxidative stress, and multiple aging hallmarks in essential organs including the liver, heart, and kidneys [142]. Furthermore, GlyNAC supplementation in old mice improved diastolic blood pressure function while NAC supplementation alone did not improve cardiac function [143].

### 7.4 L-theanine

L-theanine is the most predominant, non-proteinogenic amino acid available in green tea [144]. L-theanine also referred to as N-ethyl-L-glutamine, can be chemically synthesized from pyrrolidonecarboxylic acid and aqueous ethylamine. Studies have revealed the health benefits of L-theanine consumption include improvements in brain and gastrointestinal function, antihypertensive effects, cancer prevention, immune function improvement, improvement in mood, and reduction of stress and anxiety-like symptoms [109, 145].

The consumption of L-theanine is well tolerated and no significant adverse side effects have been reported in animal studies [109] and in humans [110, 146]. After oral administration, L-theanine is readily absorbed through the intestine into the bloodstream and transported to the main body organs and the maximum plasma concentration of L-theanine occurred 0.8 hours after 100 mg of capsule and tea administration [147]. The supplementation of 200 and 400 mg/day of L-theanine may be optimum to reduce stress and anxiety in people with acutely stressful situations [110]. The US Food and Drug Administration (FDA) recommends a total daily consumption of L-theanine should not exceed 1200 mg [147].

Studies investigating the effect of L-theanine on health outcomes in postmenopausal women are limited, but to the best of our knowledge, no studies have been conducted to investigate the effect of L-theanine on endothelial function and oxidative stress in postmenopausal women. L-theanine (200 mg) has been proposed to improve overall sleep quality (improvements in sleep quality and sleep efficiency) in all age groups [148]. Improvements in sleep quality may be due to the L-theanine effects on relaxation, changes in the autonomic nervous system (increased parasympathetic tone), regulation of neurotransmitters, and inhibition of excitatory neurons [148]. However, a pilot randomized clinical trial with healthy individuals found that blood pressure and heart rate were not reduced and there was no parasympathetic interaction after the consumption of L-theanine incorporated in a food matrix [106]. A clinical trial found that a daily dose of 400 mg of L-theanine was safe and improved several aspects of sleep quality in boys diagnosed with Attention-deficit hyperactivity disorder [149].

A daily dose of 200–400 mg of L-theanine for up to 8 weeks has been reported to be safe and improve the anxiolytic and anti-stress effects in both acute and chronic situations thus, more clinical trials are recommended to investigate the effects of chronic supplementation [150]. Another study was unable to confirm the effect of L-theanine supplementation for the treatment of generalized anxiety disorder but reported that supplementation improved sleep satisfaction and reduced sleep disturbances in individuals with mild insomnia [151]. Scores for stress-related symptoms (depression, anxiety-trait, and sleep) were reduced and scores for cognitive function (verbal fluency and executive function) improved after 4 weeks of L-theanine supplementation. However, sleep quality issues were worse after L-theanine supplementation compared with the placebo [152]. A clinical trial conducted with supplementation of green tea extract has significantly reduced circulating TC and LDL-C in postmenopausal women [153].

**Fig. 3.**
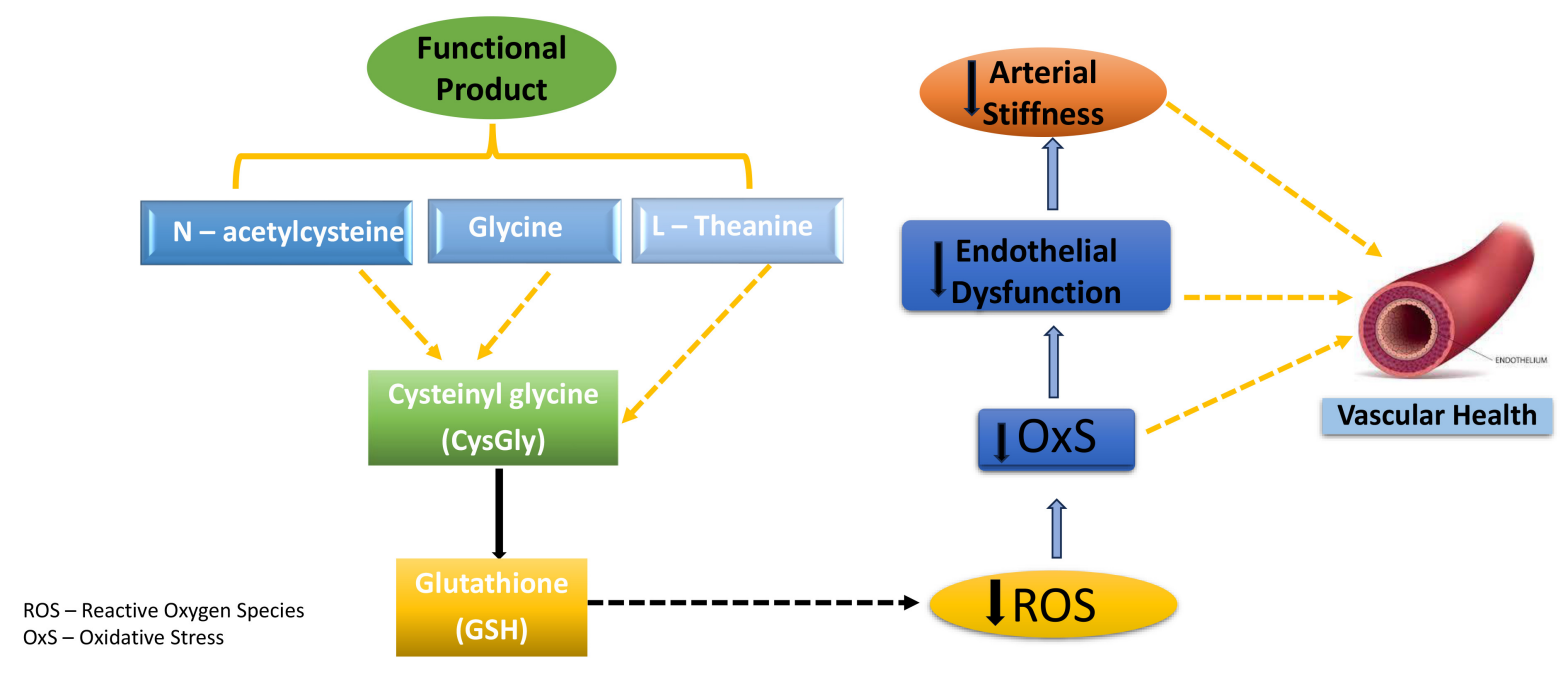
**Potential mechanism of action of N-acetylcysteine, Glycine, and L-theanine in preservation of vasculature and cardiovascular health in humans**.

Findings from human trials and animal studies have support the proposed antioxidant and anti-inflammatory activity of NAC, Glycine, and L-theanine. Thus, a functional beverage formulated with NAC, Glycine, and L-theanine may promote GSH synthesis. As previously mentioned, GSH is a tripeptide composed of three amino acids (cysteine, glycine, and glutamic acid) that may be supplied by the proposed nutraceuticals. Fig. 3 illustrates the potential mechanism of action of NAC, Glycine, L-theanine in preservation of vasculature and cardiovascular health in humans [105, 116, 137, 138]. However, the supplementation studies in postmenopausal women investigating the effects of these three amino acids independently or in combination on the cardiovascular health are scarce. Future studies are required to determine whether these amino acids, in isolation or in various combinations, are able to preserve the vasculature and cardiovascular health of postmenopausal women.

## 8. Conclusions 

The risk of developing cardiovascular and psychological health issues is increased with the menopause transition. Functional food products and mixtures that have high antioxidant and anti-inflammatory activity are proposed as novel strategies to decrease the symptoms and adverse health conditions in postmenopausal women. There is a need for research focusing on the effectiveness of antioxidant and anti-inflammatory products and substances in the management of cardiovascular health in postmenopausal women. Moreover, therapeutic interventions are important to reduce the adverse health effects of menopause transition and maintain the overall health of postmenopausal women. Furthermore, future studies determining the concentration of the compound, duration of administration, and acute and long-term outcomes of the supplements are needed.

## Abbreviations

CVD, Cardiovascular disease; MAFLD, Metabolic dysfunction-associated fatty liver disease; MetS, Metabolic syndrome; E2, Estradiol; FSH, Follicle stimulating hormone; T2DM, Type 2 diabetes mellitus; NO, Nitric oxide; eNOS, Endothelial nitric oxide synthase; ROS, Reactive oxygen species; DNA, Deoxyribonucleic acid; DHEA, Dehydroepiandrosterone; DHEAS, Dehydroepiandrosterone sulphate; DHT, Dihydrotestosterone; SWAN, The Study of Women’s Health Across the Nation; HRT, Hormone replacement therapy; GSH, Glutathione; FMD, Flow-mediated dilation; LA, Lipoic acid; DHA, Docosahexaenoic acid; EPA, Eicosapentaenoic acid; NAC, N – acetylcysteine; GlyNAC, Glycine and N-acetylcysteine.
